# A technique modification in the cannulation to the innominate artery in the surgical treatment of aortic dissection

**DOI:** 10.1186/s13019-015-0394-7

**Published:** 2015-12-30

**Authors:** Yang Yu, Tianxiang Gu, Enyi Shi, Lei Yu, Chun Wang

**Affiliations:** Department of Cardiac Surgery, First Affiliated Hospital, China Medical University, Shenyang, 110001 China

**Keywords:** Aortic dissection, CPB components

## Abstract

**Background:**

A modified cannulation strategy to innominate artery was introduced which differs from traditional cannulation method used in aortic surgeries.

**Case presentation:**

Four patients suffering from aortic dissections with or without other cardiac diseasees underwent surgical treatment by using the modified canulation technique. All patients had an uneventful perioperative period and discharged from the hospital without any complications.

**Conclusions:**

Innominate artery cannulation using the modified cannula with “a hole in the back” is an easy and effective strategy for arch surgery.

## Background

**Fig. 1:**
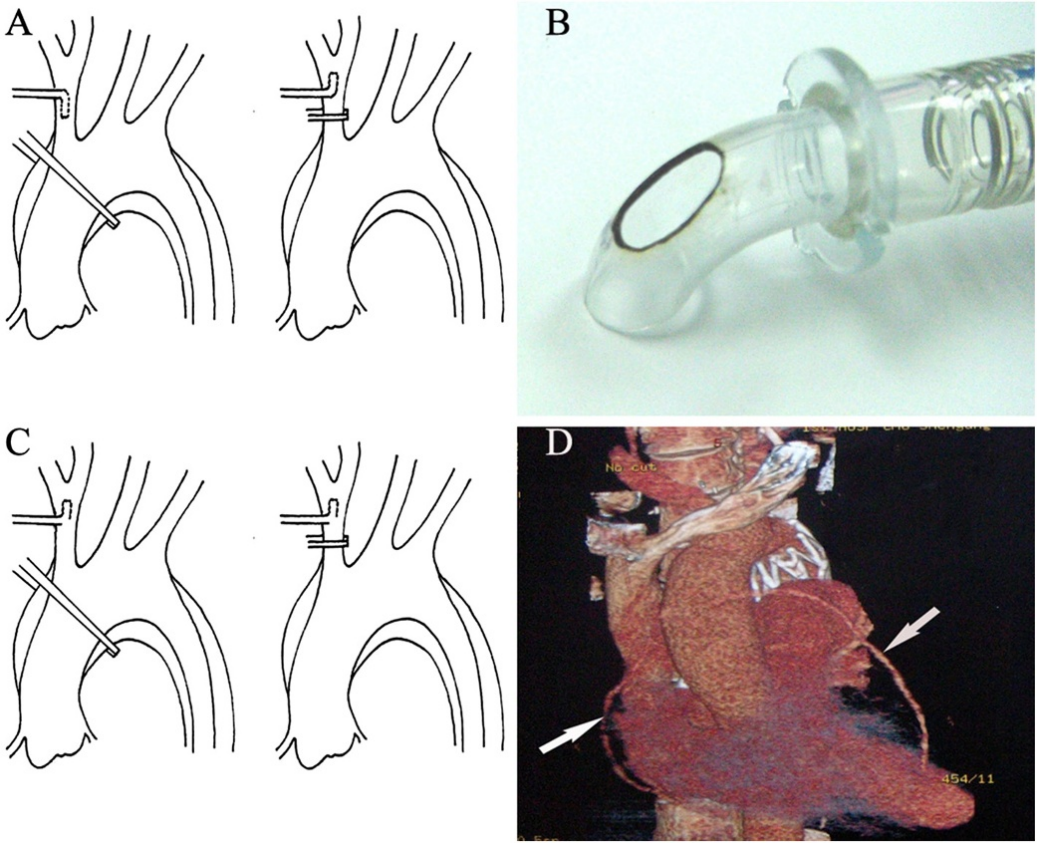
**a**: Diagram of innominate artery cannulation using normal cannula. **a**, For cooling and rewarming, the tip of the cannula is pointing toward the arch and the crossclamp is placed at the ascending aorta. **b**, For selective antegrade cerebral perfusion, the tip of the cannula is oriented toward the head and the crossclamp is placed proximal to the site of cannulation of the innominate artery. **b**: Photograph of modified innominate artery cannula with “a hole in the back”. **c**: Diagram of innominate artery cannulation using modified cannula. **a**, For cooling and rewarming, the crossclamp is placed at the ascending aorta. **b**, For selective antegrade cerebral perfusion, the crossclamp is placed proximal to the site of cannulation of the innominate artery. **d**: Postoperative computerized tomography angiography of a patient with type B dissection and coronary artery disease. Arrows show the venous graft for coronary artery bypass graft. The mental stent of stented elephant graft is located inside the descending aorta

Innominate artery cannulation has been widely accepted in arch surgery since it was reported by Banbury and Cosgrove [1, 2]. In our department the technique has been used in the surgical treatment of Stanford A aortic dissections. The tip of the cannula was rotated during the switch between system perfusion and selective antegrade cerebral perfusion (SCP) (Fig. 1a) [3]. To simplify such procedure, a modification to the cannula was made and the clinical experience is presented.

## Case presentation

There were four patients in the series. Two patients of type B dissection combined with coronary artery disease received stented elephant trunk implantation and CABG; one patient of type B dissection combined with aortic valve insufficiency received stented elephant trunk implantation by an open aortic manner and tissue valve replacement; one patient of type A dissection received ascending aorta and hemiarch replacement combined with stented elephant trunk implantation.

A median sternotomy was performed, the innominate artery was mobilized to the bifurcation as well as left common carotid artery and left subclavian artery. After heparinzation, double purse-string sutures were made at the innominate artery. Then the innominate artery was stabbed and a home-made 22F or 24F wire-reinforced flexible short-tipped cannula with “a hole in the back” (Fig. 1b) was introduced. The hole was made at the bend opposite to the tip with a similar area as the normal opening of the cannula. Venous return was established with a 2-stage cannula introduced via the right atrium. When the core temperature was cooled to about 32 °C, the ascending aorta was clamped, cardioplegia was perfused antegradely. The proximal procedures: distal anastomosis for CABG, aortic valve replacement or ascending aorta replacement, were performed. During the proximal procedure the core temperature was decreased sequentially to 26 ~ 28 °C, then the circulation arrest was induced and a clamp was applied proximal to the innominate artery cannula (Fig. 1c). The antegrade SCP was induced with a rate of 5 to 10 ml/kg/min with maintaining the right radial artery pressure at 40 ~ 50 mmHg. Such a pressure was almost the same as what was maintained during system perfusion. Then the arch was transected and the returning blood from left common carotid artery was well observed. A self-expandable stent vessel prosthesis with a length of 8 to 10 cm was inserted into the descending aorta. The proximal end of the stent was positioned just distal to the opening of the left subclavian artery and proximal free edge was attached to the aorta with a continuous suture. The incision at the aortic arch was closed. Then the clamp on innominate artery was removed and the ascending aorta was occluded again. Systemic perfusion was restored gently. Then the rewarming progress was induced.

All patients had an uneventful perioperative period and discharged without any complication. Permanent neurologic dysfunction and paraplegia did not occur in the patients. Postoperative CTA of a patient with type B dissection and coronary artery disease is shown in Fig. 1d.

## Conclusions

Innominate artery cannulation is well accepted in aortic arch surgery, for it can be used during total CPB as well as SCP. Compared with traditional technique using right axillary artery cannulation to induce SCP, the innominate artery cannulation processes several advantages: First, a second incision to gain access to the axillary artery can be avoided. Second, the innominate artery is usually larger than the right subclavian or axillary arteries, total CPB flow can be easily achieved without high pump pressures, also the cannulation can be easier. Third, the SCP pressure can be monitored by a routine right radial arterial line. Finally, the risk for brachial plexus injury or right axillary artery injury can be avoided [4–6]. During the aortic procedure involving the arch, the circulation arrest and SCP are often induced by an added subclavian incision and the cannulation in the right axillary artery. Our centre had reported a series of surgical treatment for the patients with type A dissections using innominate cannulation and the result is encouraging. Based on this technique, a modification to the cannula was made: a hole with a similar size as the normal opening was made in the back of the tip. During systemic perfusion, blood can be perfused from the hole to the aorta. When the innominate artery was occluded, SCP can be induced through the normal opening of the cannula. Therefore, the rotation of the tip during switch between system perfusion and SCP was avoided [7, 8]. The surgical procedure for arch was further simplified. No permanent neurologic dysfunction and paraplegia was detected in the series. The current results indicted that both system perfusion and SCP can be performed by using the modified cannula to innominate artery without complications and such technique is an easy and effective strategy for arch surgery.

However the cannulation technique itself has some limitations: 1. The exposure of the innominate artery must long enough so that the clamp could be applied to the proximal site of the innominate artery without touching the cannula; 2. For the patients with the brachiocephalic vessels especially the innominate artery affected by dissections, this technique should be abandoned. 3. If atherosclerosis, stenosis, or plaques was found in the innominate artery, the cannulation should be cautiously.
